# Association Between Ultra-Processed Food Consumption and Frailty in Korean Older Adults

**DOI:** 10.3390/nu18193135

**Published:** 2026-09-23

**Authors:** Hyewon Lee, Kyong Park

**Affiliations:** Department of Food and Nutrition, Yeungnam University, 280 Daehak-ro, Gyeongsan 38541, Republic of Korea; lhw0072@yu.ac.kr

**Keywords:** ultra-processed foods, frailty, older adults, Korea

## Abstract

Background/Objectives: Population aging is accelerating worldwide, and frailty has emerged as an important health concern among older adults. Although ultra-processed food (UPF) consumption has been associated with a range of adverse health outcomes, evidence on its relationship with frailty among Korean older adults remains limited. This study examined the cross-sectional association between the proportion of total energy intake derived from UPFs and frailty among Korean adults aged 65 years or older. Methods: We analyzed data from 9861 adults aged ≥65 years who participated in the 2014–2019, 2022, and 2024 Korea National Health and Nutrition Examination Survey cycles, in which the variables required to assess frailty were available. Dietary intake was assessed using a 24-h dietary recall, and foods were classified according to the NOVA food classification system. Frailty was defined using a modified Fried frailty phenotype. Survey-weighted multivariable logistic regression models were used to examine associations between UPF energy contribution and frailty, and restricted cubic spline models were used to evaluate the dose–response relationship. Results: In the fully adjusted model, participants in the highest quartile of UPF energy contribution had 31% higher odds of frailty than those in the lowest quartile (odds ratio [OR] = 1.31, 95% confidence interval [CI]: 1.08–1.59; *p* for trend = 0.002). Among the individual frailty components, participants in the highest quartile also had higher odds of low physical activity (OR = 1.31, 95% CI: 1.13–1.51; *p* for trend = 0.001). Restricted cubic spline analysis showed no evidence of a nonlinear association between UPF energy contribution and frailty (*p* for nonlinearity = 0.8). Conclusions: Higher UPF consumption was positively associated with frailty among Korean older adults, particularly with low physical activity. These findings highlight the relevance of UPF consumption to frailty in an older population with relatively low levels of UPF intake.

## 1. Introduction

Population aging is accelerating worldwide. The global population aged 60 years or older is projected to increase from 1.0 billion in 2019 to 1.4 billion by 2030 and 2.1 billion by 2050 [1]. Korea is experiencing particularly rapid population aging; the proportion of adults aged 65 years or older exceeded 20.3% in 2025, marking its transition to a super-aged society, and is projected to reach 47.5% by 2070 [2]. As longevity increases, preventing frailty becomes a public health priority for preserving functional independence and quality of life.

Frailty is a common geriatric syndrome characterized by reduced physiological reserve and increased vulnerability to adverse health outcomes, including falls, disability, hospitalization, and mortality [3]. An analysis of data from the Korea National Health and Nutrition Examination Survey (KNHANES) using a frailty index found that 23.1% of adults aged 65 years or older were classified as frail and 32.7% as prefrail in 2020 [4]. In an analysis of the 2023 National Survey of Older Koreans, 4.5% of adults aged 65 years or older were classified as frail and 32.3% as prefrail using the K-FRAIL scale [5]. The development of frailty is multifactorial, involving nutritional, genetic, metabolic, environmental, and lifestyle-related factors, as well as acute and chronic diseases [6,7]. Among these factors, diet is of particular interest because it is potentially modifiable and provides the energy and nutrients needed to maintain muscle mass and physical function, which are closely related to frailty [8]. Previous studies have shown that poorer overall diet quality is associated with a greater likelihood of frailty in older adults [7,9].

Ultra-processed foods (UPFs), as defined by the NOVA food classification system, are industrial formulations typically produced from highly processed food substances and additives, such as sweeteners, colorants, and emulsifiers [10]. UPFs are often characterized by higher contents of added sugars, sodium, saturated fat, and trans fat and lower contents of dietary fiber and micronutrients [10]. Their availability and consumption have increased substantially across countries with diverse levels of economic development, contributing to a global shift toward diets with a greater reliance on highly processed foods [11]. Higher UPF consumption may contribute to frailty through chronic inflammation, oxidative stress, and metabolic dysregulation. These processes may promote muscle catabolism and impair muscle function, thereby reducing physiological reserve and increasing vulnerability to frailty [12]. Nevertheless, UPFs comprise a heterogeneous category of foods with diverse nutritional compositions and processing characteristics, and their associations with health outcomes may vary across specific UPF subgroups [13].

Epidemiological studies conducted in Spain [14], Italy [15], the United States [16,17], Australia [18], and Iran [19] have generally reported higher risks or prevalence of frailty among older adults with greater UPF consumption. However, few studies have examined this association in East Asian older adults, whose dietary patterns and UPF sources may differ from those of previously studied populations. In Korea, the UPF sources may include culturally specific products, such as industrially formulated versions of traditional fermented sauces and condiments that meet the NOVA criteria for UPFs. Although UPF consumption among Korean adults aged 60 years or older remains lower than that among younger adults, it has increased over time [20].

To address the limited evidence in East Asian older adults, this study examined whether a higher proportion of total energy intake derived from UPFs was associated with frailty among Korean adults aged 65 years or older, using nationally representative cross-sectional data from KNHANES. As a secondary objective, we examined associations with individual frailty components to characterize patterns that may not be apparent from the overall frailty classification.

## 2. Materials and Methods

### 2.1. Data Source and Participants

KNHANES is an ongoing, nationally representative, cross-sectional survey that assesses health behaviors, chronic conditions, and dietary intake among the Korean population through health interviews, health examinations, and nutrition surveys [21].

This study was a pooled secondary analysis of data from repeated cross-sectional KNHANES surveys from 2014–2019, 2022, and 2024, rather than a longitudinal analysis of the same individuals. These survey years were selected because they included the measures required to assess frailty, including handgrip strength and the EuroQol-5 Dimensions (EQ-5D) [21]. Handgrip-strength data were unavailable for 2020–2021 because measurements were limited during the COVID-19 pandemic and nationally representative estimates could not be produced. The 2023 survey was excluded because the EQ-5D was not administered that year [21]. For the descriptive analysis of temporal trends in UPF consumption, all KNHANES survey years from 2014 through 2024 with available dietary intake data were included, regardless of the availability of variables required for frailty assessment.

Among 54,481 participants from the selected survey years, we excluded those aged younger than 65 years (*n* = 42,011), those with missing survey sample weights (*n* = 320), those with missing data required to determine frailty status (*n* = 2155), and those with implausible total energy intake (<500 or >5000 kcal/day; *n* = 134). The final sample for the primary frailty analyses comprised 9861 participants aged 65 years or older (Supplementary Figure S1). Annual sample sizes for the primary frailty analyses and the UPF temporal-trend analysis are provided in Supplementary Table S3.

Written informed consent was obtained from all KNHANES participants. The surveys used in this study were approved by the Institutional Review Board (IRB) of the Korea Disease Control and Prevention Agency (approval numbers: 2013-12EXP-03-5C, 2018-01-03-P-A, 2018-01-03-C-A, 2018-01-03-4C-A, and 2022-11-16-R-03). For 2015–2017, KNHANES was conducted without separate IRB review in accordance with the recommendation of the Korea Disease Control and Prevention Agency IRB [21].

### 2.2. Demographic and Lifestyle Information

Information on sex, age, educational level, and household income was obtained through interviewer-administered questionnaires [21]. Alcohol consumption and smoking status were assessed using self-administered questionnaires, whereas height and weight were measured during the health examination. Educational level was categorized as elementary school or lower, middle school, high school, or college or higher. Household income was classified as low, middle, or high based on the quartiles of average monthly equivalized household income provided in the KNHANES; the middle category combined the original lower-middle and upper-middle categories of equivalized monthly household income. Smoking status was categorized as current smokers or non-current smokers based on current smoking behavior. Participants who reported any alcohol consumption during the previous year were classified as drinkers, whereas those reporting no alcohol consumption were classified as non-drinkers. The body mass index (BMI) was calculated as weight in kilograms divided by height in meters squared (kg/m^2^).

### 2.3. Dietary Intake Assessment and Definition of Ultra-Processed Foods

Dietary intake was assessed by trained interviewers using a 24-h dietary recall. From 2014 through 2019, interviewers visited participants’ homes and collected information on foods and beverages consumed on the previous day. In 2022 and 2024, dietary intake was assessed at mobile examination centers for foods and beverages consumed two days before the interview [21].

UPFs were identified according to the NOVA food classification system developed by Monteiro et al. [22,23]. NOVA classifies foods into four groups according to the extent and purpose of industrial processing: unprocessed or minimally processed foods (Group 1), processed culinary ingredients (Group 2), processed foods (Group 3), and ultra-processed foods (Group 4). Detailed classification criteria have been described elsewhere [22,23].

Mixed dishes were classified as single food items based on the final prepared product, without disaggregating recipes into individual ingredients before applying NOVA. For items with classification uncertainty, particularly for culturally specific foods and products with limited information on processing, we reviewed classifications reported in previous KNHANES-based studies (Supplementary Table S1) and relevant evidence supporting individual classification decisions. This process was undertaken to support consistent application of the NOVA criteria within the Korean dietary context. For fermented sauces, classification was based on ingredients and manufacturing methods. Products clearly identified as traditionally home-prepared were not classified as UPFs. When preparation details were insufficient, additional product information was reviewed. Commercially produced sauces were classified as UPFs when their ingredients or manufacturing processes indicated ultra-processing under NOVA, including acid hydrolysis where documented. Two investigators then independently classified foods according to the NOVA criteria, and any discrepancies were resolved by consensus. The resulting UPF items were grouped into four major categories, with the specific foods and beverages included in each category presented in Supplementary Table S2.

### 2.4. Definition of Frailty

Frailty was assessed based on the Fried frailty phenotype, with modifications to accommodate the variables available in KNHANES, as described in previous KNHANES-based studies [3,24,25,26]. Frailty was assessed using five components: unintentional weight loss, weakness, slowness, exhaustion, and low physical activity. For low physical activity, we selected a criterion that could be consistently applied across the included survey years and that was consistent with previous studies [24,27,28,29,30,31,32]; alternative definitions were examined in sensitivity analyses.

Unintentional weight loss was defined as a self-reported unintentional loss of at least 3 kg during the previous year. Weakness was assessed using a T.K.K. 5401 digital handgrip dynamometer (Takei Scientific Instruments Co., Ltd., Tokyo, Japan). Grip strength was measured two or three times for each hand, depending on the survey year, and the mean value for the dominant hand was calculated. The mean was selected to align with the original Fried protocol, which averaged three dominant-hand measurements obtained with maximal effort. For participants who reported using both hands equally, the mean of all measurements from both hands was used [33,34]. Weakness was defined by applying the sex-specific cutoffs proposed by the 2019 Asian Working Group for Sarcopenia (<28 kg in men and <18 kg in women) to the mean grip strength [35].

Because direct measures of gait speed and exhaustion were not available consistently in KNHANES, these components were operationalized using available questionnaire items. Slowness was defined using the EQ-5D mobility item and was considered present when participants reported either some problems with walking or being confined to bed. Exhaustion was defined using a perceived-stress question and was considered present when participants reported feeling very or extremely stressed. Low physical activity was assessed using the Global Physical Activity Questionnaire and was defined using sex-specific cutoffs corresponding to the lowest 20% of physical activity based on a national survey of Korean older adults (<494.65 kcal/week for men and <283.50 kcal/week for women) [36].

Frailty status was determined only for participants with complete data on all five components. Each component was assigned 1 point when the criterion was met and 0 otherwise, yielding a total score ranging from 0 to 5. Participants were classified as frail (≥3 points), prefrail (1–2 points), or robust (0 points). For the analysis, frailty was dichotomized as frail (≥3 points) versus non-frail (0–2 points).

### 2.5. Statistical Analysis

All analyses accounted for the complex sampling design of KNHANES by incorporating survey weights, stratification variables, and primary sampling units. For the pooled frailty analyses, the annual KNHANES weights were divided by eight, corresponding to the eight included survey years (2014–2019, 2022, and 2024), each covering one full year.

UPF energy contribution (%) was calculated using the following equation:

UPF energy contribution (%) = [energy intake from NOVA Group 4 foods (kcal/day)/total daily energy intake (kcal/day)] × 100.

All foods and beverages classified as UPFs were included regardless of their energy content, with no minimum energy threshold applied. Each item’s reported energy value was used in the calculation; zero-energy items contributed zero to the UPF energy numerator.

Overall UPF energy contribution was categorized into quartiles for the primary analyses. For analyses of specific UPF categories, a high proportion of zero intakes in some categories precluded conventional quartile classification. Therefore, participants were classified into four intake groups using category-specific cutoffs based on the observed intake distributions.

Temporal trends in UPF energy contribution were assessed using survey-weighted linear regression, with calendar year entered as a continuous variable and adjustment for age and sex. Participant characteristics across quartiles of UPF energy contribution were compared using survey-weighted linear regression for continuous variables and design-adjusted chi-square tests for categorical variables. Survey-weighted multivariable logistic regression models were used to estimate odds ratios (ORs) and 95% confidence intervals (CIs) for the associations of UPF energy contribution with frailty and each of its five components. Overall frailty was the primary outcome, and analyses of the five individual frailty components were prespecified secondary analyses. No adjustment for multiple comparisons was applied; therefore, component-specific findings should be interpreted cautiously in view of multiple testing. Tests for linear trend were conducted by assigning the median UPF energy contribution of each quartile to participants in that quartile and modeling this value as a continuous variable.

Potential confounders were selected based on the previous literature and their established or plausible associations with UPF intake and frailty [16,17,32,37]. Three models with increasing levels of covariates adjustment were fitted: Model 1 was unadjusted; Model 2 was adjusted for age and sex; and Model 3 was additionally adjusted for household income, educational level, survey year, alcohol consumption, smoking status, BMI, and total energy intake.

Subgroup analyses were conducted according to sex, household income, educational level, smoking status, alcohol consumption, BMI, and total energy intake. Potential effect modification was evaluated by including multiplicative interaction terms between UPF energy contribution and each potential modifier.

Restricted cubic spline models with knots at the 25th, 50th, and 75th percentiles of UPF energy contribution were used to examine the shape of the dose–response association between UPF energy contribution and frailty, with adjustment for the same covariates as in Model 3. Sensitivity analyses were performed using alternative definitions of low physical activity: <2 h/week of moderate-intensity activity, <1 h/week of vigorous-intensity activity, and <2 h/week of walking activity. An additional sensitivity analysis excluded industrially manufactured fermented sauces from the UPF classification and re-estimated the association with frailty using the same multivariable models (Supplementary Table S6).

All statistical analyses were performed using SAS version 9.4 (SAS Institute, Cary, NC, USA) and Stata/SE 13 (StataCorp, College Station, TX, USA). All statistical tests were two-sided, and a *p* value < 0.05 was considered statistically significant.

## 3. Results

### 3.1. Trends in UPF Consumption

Figure 1 shows the annual weighted mean UPF energy contribution among Korean adults aged 65 years or older from 2014 to 2024, with detailed annual estimates provided in Supplementary Table S3. The temporal-trend analysis included all survey years with available dietary intake data during this period, including 2020, 2021, and 2023, which were excluded from the primary frailty analyses. The weighted mean UPF energy contribution increased from 11.10% in 2014 to 12.52% in 2024, corresponding to an increase of 1.42 percentage points. Survey-weighted linear regression adjusted for age and sex estimated an average annual increase of 0.16 percentage points in UPF energy contribution (95% CI: 0.09–0.24; *p* for trend <0.001).

### 3.2. General Characteristics of Participants According to UPF Energy Contribution

Table 1 presents the characteristics of participants according to quartiles of UPF energy contribution. Mean UPF energy contribution was 1.24% in Q1 and 29.26% in Q4. The corresponding median (interquartile range) values were 1.08% (0.29–2.12%) and 25.39% (20.11–34.54%), respectively. The observed ranges were 0.00–3.14% in Q1 and 16.29–99.62% in Q4. The distributions of sex, household income, educational level, alcohol consumption, and smoking status differed significantly across quartiles (all *p* < 0.05). Total energy intake also differed across quartiles, whereas age (*p* = 0.227) and BMI (*p* = 0.204) did not.

### 3.3. Association Between UPF Energy Contribution and Frailty

In the analytic sample of 9861 participants, the overall survey-weighted proportions classified as frail, prefrail, and robust were 16.20%, 53.44%, and 30.36%, respectively. Table 2 presents the ORs and 95% CIs for frailty and its individual components across quartiles of UPF energy contribution. The survey-weighted prevalence of frailty was 15.73% in Q1 and 17.10% in Q4, corresponding to an unadjusted absolute difference of 1.37 percentage points. In the fully adjusted model (Model 3), participants in the highest quartile (Q4) had 31% higher odds of frailty than those in the lowest quartile (Q1) (OR = 1.31, 95% CI: 1.08–1.59; *p* for trend = 0.002). Among the covariates added individually to Model 2, educational level produced the largest increase in the Q4 versus Q1 OR, from 1.20 (95% CI: 1.00–1.44) to 1.36 (95% CI: 1.13–1.63). Adding other individual covariates resulted in relatively modest changes (Supplementary Table S4). Among the individual frailty components, only low physical activity showed a significant positive trend across quartiles of UPF energy contribution. Participants in Q4 had 31% higher odds of low physical activity than those in Q1 (OR = 1.31, 95% CI: 1.13–1.51; *p* for trend = 0.001). No significant trends were observed for unintentional weight loss, weakness, slowness, or exhaustion. The association between UPF energy contribution and frailty did not differ significantly by sex, household income, educational level, smoking status, alcohol consumption, BMI, or total energy intake (all *p* for interaction >0.05; Supplementary Figure S2).

Sensitivity analyses using alternative definitions of low physical activity yielded mixed results (Supplementary Table S5). The association with frailty was attenuated when the low physical activity was defined based on moderate- or vigorous-intensity activity (Q4 vs. Q1: OR = 1.08, 95% CI: 0.92–1.27; *p* for trend = 0.225), whereas it remained significant when defined based on walking time (OR = 1.33, 95% CI: 1.11–1.61; *p* for trend = 0.001).

A separate sensitivity analysis excluding industrially manufactured fermented sauces from the UPF classification yielded findings consistent with the primary analyses (Supplementary Table S6).

### 3.4. Associations Between Specific UPF Categories and Frailty

The distribution of consumers and energy intake across specific UPF categories is presented in Supplementary Table S7. Associations between specific UPF categories and frailty are shown in Figure 2. In the fully adjusted model, participants in the highest intake group for packaged breads, breakfast cereals, sweet or savory snacks, and desserts had 31% higher odds of frailty than those in the lowest intake group (OR = 1.31, 95% CI: 1.07–1.61). Similarly, those in the highest intake group for artificially sweetened and sugar-sweetened beverages had 22% higher odds of frailty (OR = 1.22, 95% CI: 1.03–1.44). The corresponding ORs were 0.98 (95% CI: 0.79–1.21) for convenience foods and animal-based processed foods and 0.86 (95% CI: 0.72–1.03) for sauces, condiments, and spreads.

### 3.5. Dose–Response Relationship Between UPF Energy Contribution and Frailty

Figure 3 shows the restricted cubic spline analysis of the association between UPF energy contribution and frailty. In the fully adjusted model, the spline curve suggested a gradual increase in the odds of frailty with increasing UPF energy contribution. There was no evidence of a nonlinear association (*p* for nonlinearity = 0.8).

## 4. Discussion

In this nationally representative cross-sectional study of Korean adults aged 65 years or older, higher UPF energy contribution was associated with higher odds of frailty after multivariable adjustment (Q4 vs. Q1: OR = 1.31, 95% CI: 1.08–1.59). The spline analysis suggested an overall positive pattern, with no evidence of nonlinearity. Among the five frailty components, low physical activity was significantly associated with UPF energy contribution. In analyses according to specific UPF categories, higher intake of packaged breads, breakfast cereals, sweet or savory snacks, and desserts and of artificially sweetened and sugar-sweetened beverages was associated with frailty.

Our findings are broadly consistent with previous studies of older adults conducted in Spain, Italy, the United States, Australia, and Iran [14,15,16,17,18,19]. Although Konieczynski et al. reported no significant association between UPF consumption and frailty [38], differences across studies may reflect variation in the types of foods contributing to UPF intake, the application of the NOVA classification, methods used to assess frailty, and characteristics of the study populations. A systematic review including cross-sectional and cohort studies similarly reported an association between higher UPF consumption and frailty among adults older than 60 years [39]. More recently, a dose-response analysis within a recent meta-analysis reported a 3% higher risk of frailty per 100-g/day increment in UPF intake [40].

The present study extends this evidence to Korean older adults, an understudied East Asian population with a dietary context and sources of UPFs that may differ from those examined in previous studies. UPF consumption in Korean older adults is also lower than that observed in younger Korean adults [20], making this population particularly informative for examining whether the association with frailty is evident at lower levels of UPF intake. In addition, our study examined UPF intake as a proportion of total energy intake and explored specific UPF categories and individual components of the frailty phenotype, providing a more detailed characterization of the association. Frail older adults may rely more on convenient or ready-to-eat UPFs because of difficulties with shopping or meal preparation, impaired mobility, or fatigue. Thus, reverse causation may partly explain the observed association between UPF energy contribution and overall frailty.

Among the five frailty components, low physical activity showed a significant positive association with UPF energy contribution. Although the estimated associations remained positive in sensitivity analyses, their magnitude and statistical significance of the association with frailty varied when alternative definitions of low physical activity were used to define frailty. UPF consumption may plausibly contribute to poorer physiological resilience, while frailty and functional decline may also increase reliance on convenient and readily available UPFs. Given the cross-sectional design, the direction of this relationship cannot be determined. The co-occurrence of higher UPF intake and low physical activity may reflect a broader pattern of health-related behaviors. Socioeconomic circumstances affecting food access and meal preparation may also influence UPF intake and may not be fully captured by adjustment for household income and education.

The category-specific analyses provided further insight into the overall association between UPF consumption and frailty. Higher intake of packaged breads, breakfast cereals, sweet or savory snacks, and desserts, as well as artificially sweetened and sugar-sweetened beverages, was associated with frailty. These categories include many commercially formulated foods and beverages that are often characterized by refined carbohydrates, added sugars, sodium, and high energy density, although their nutritional profiles may vary across individual products [10]. In contrast, sauces, condiments, and spreads showed an inverse, although not statistically significant, association with frailty. This category warrants particular consideration in the Korean dietary context because it included commercially manufactured versions of traditional fermented condiments, such as doenjang, soy sauce, gochujang, and ssamjang. These products were classified as UPFs because of industrial processing and the use of additives rather than fermentation itself. Traditionally prepared fermented condiments may differ substantially from their industrially manufactured counterparts in ingredients, processing methods, and biological properties. Previous research among Korean older adults has reported an inverse association between traditional fermented condiment intake and prefrailty [41], and fermentation may generate bioactive compounds with potentially beneficial physiological effects [42]. The inverse association observed in the present study was not statistically significant and does not provide evidence of a protective effect. Findings regarding traditionally prepared fermented foods cannot be directly extrapolated to the commercially produced sauces classified as UPFs in this study. Nevertheless, this finding highlights the heterogeneity of foods encompassed by the NOVA UPF category and the importance of considering how culturally specific foods are produced and consumed. In the Korean dietary context, applying NOVA to commercially produced fermented sauces, mixed dishes, and industrially manufactured versions of traditional foods requires careful consideration of their ingredients and processing methods. Foods classified as UPFs may differ across dietary cultures in nutritional composition and patterns of consumption. These differences should be considered when interpreting associations between UPF intake and frailty across populations. Thus, both the amount of UPF intake and the specific food sources contributing to that intake may be relevant when examining its relationship with frailty across populations.

Several potential pathways may help explain the observed association between UPF consumption and frailty. Diets high in UPFs have been associated with systemic inflammation and oxidative stress, both of which may contribute to age-related functional decline [15,43]. Chronic low-grade inflammation may impair the maintenance of skeletal muscle by increasing myostatin activity and suppressing insulin-like growth factor-1 signaling involved in protein synthesis [40,43]. Mechanisms related to industrial processing itself may include the effects of food additives commonly present in UPFs. Food additives used in some UPFs, including certain emulsifiers and artificial sweeteners, may alter the gut microbiota, promote intestinal inflammation, and increase intestinal permeability [44]. These changes may contribute to metabolic dysregulation and reduced physiological resilience with aging [45]. Meanwhile, diets high in UPFs tend to have poorer overall diet quality, characterized by higher intakes of added sugars, saturated fat, and sodium and lower intakes of protein, dietary fiber, and micronutrients [46,47]. In addition, greater UPF consumption may displace protein-rich or other nutrient-dense minimally processed foods from the diet [46]. These nutritional characteristics may contribute to metabolic abnormalities and an increased risk of chronic conditions, including abdominal obesity, diabetes, and cardiovascular disease, which may in turn reduce physiological reserve and increase vulnerability to frailty [13,48,49,50]. Therefore, not only factors related to industrial processing itself but also the nutritional composition and food displacement associated with UPF-rich diets may contribute to the observed association. These pathways represent potential explanations for the observed association, but their respective contributions could not be distinguished in this cross-sectional study.

The present study has several strengths. This study extends the evidence on UPF consumption and frailty to Korean older adults, a population that has been understudied in this context. The use of a large, nationally representative KNHANES sample enhances the relevance of the findings to community-dwelling Korean adults aged 65 years or older. We applied the NOVA framework to culturally specific Korean foods with reference to classifications reported in previous KNHANES-based studies. Two investigators independently classified each food item and resolved discrepancies by consensus, supporting the transparency and consistency of the classification process. In addition to overall UPF energy contribution, we examined specific UPF categories and individual frailty components and evaluated the dose–response pattern and robustness of the findings using sensitivity analyses.

Several considerations should be taken into account when interpreting these findings. First, the cross-sectional design does not allow the temporal direction of the association between UPF consumption and frailty to be determined. Second, UPF intake was assessed using a single 24-h dietary recall and therefore may not fully represent habitual intake because of day-to-day variation and recall error. In addition, our analyses did not specifically adjust for the day of the week or season of dietary intake, which may have affected UPF exposure assessment and the interpretation of temporal trends. Third, although frailty was assessed based on the Fried phenotype and approaches used in previous KNHANES studies, slowness and exhaustion were operationalized using the EQ-5D mobility and perceived-stress items rather than direct gait-speed and conventional exhaustion measures. These proxy measures may not fully capture the original Fried constructs of slowness and exhaustion. In particular, perceived stress may not adequately represent exhaustion, potentially resulting in misclassification of frailty status. The findings also varied somewhat when alternative definitions of low physical activity were applied. Finally, residual confounding by unmeasured or imperfectly measured factors cannot be excluded. Such factors may include polypharmacy, food insecurity, and social support, which were not included in the multivariable models. These considerations should be taken into account when interpreting the observed associations, particularly with respect to their temporal direction and magnitude.

## 5. Conclusions

In this cross-sectional study, higher UPF energy contribution was associated with frailty among community-dwelling Korean adults aged 65 years or older. Component- and category-specific findings should be interpreted as exploratory, particularly because the magnitude and statistical significance of the association with frailty varied across alternative operational definitions of low physical activity. For clinicians, UPF intake may be considered within a broader assessment of dietary quality and functional status. These findings highlight the potential relevance of UPF intake as a dietary marker in research on healthy aging. Prospective studies with repeated assessments of diet and frailty are needed to clarify temporal relationships before these findings can inform recommendations to reduce NOVA-defined UPF intake for frailty prevention.

## Figures and Tables

**Figure 1:**
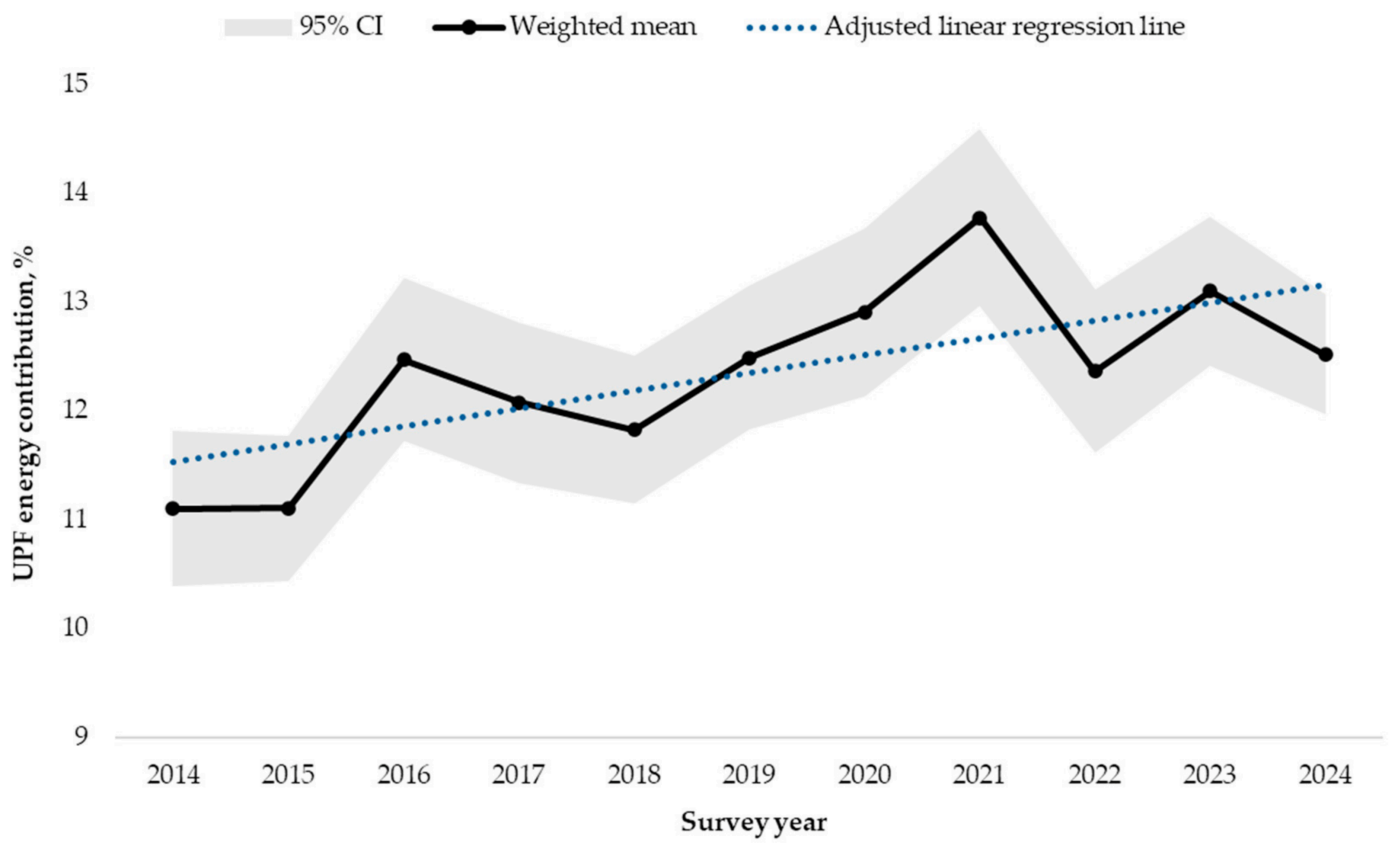
Annual weighted mean UPF energy contribution (%) among Korean adults aged 65 years or older, KNHANES 2014–2024. The solid line represents the annual weighted means, and the shaded area indicates their 95% confidence intervals. The dotted line shows the fitted temporal trend from survey-weighted linear regression adjusted for age and sex. CI, confidence interval; KNHANES, Korea National Health and Nutrition Examination Survey; UPF, ultra-processed food.

**Figure 2:**
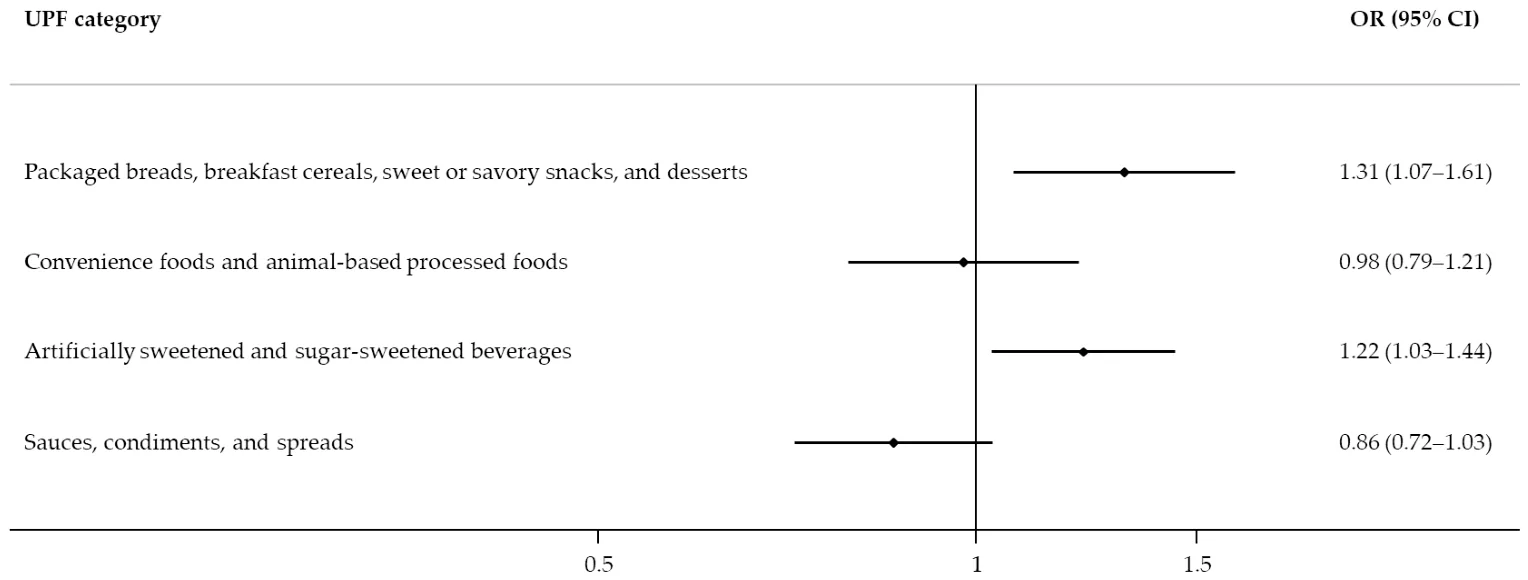
Adjusted odds ratios (95% confidence intervals) for frailty comparing the highest with the lowest intake group across specific ultra-processed food categories. Models were adjusted for age, sex, household income, educational level, survey year, alcohol consumption, smoking status, body mass index, and total energy intake. OR, odds ratio; CI, confidence interval; UPF, ultra-processed food.

**Figure 3:**
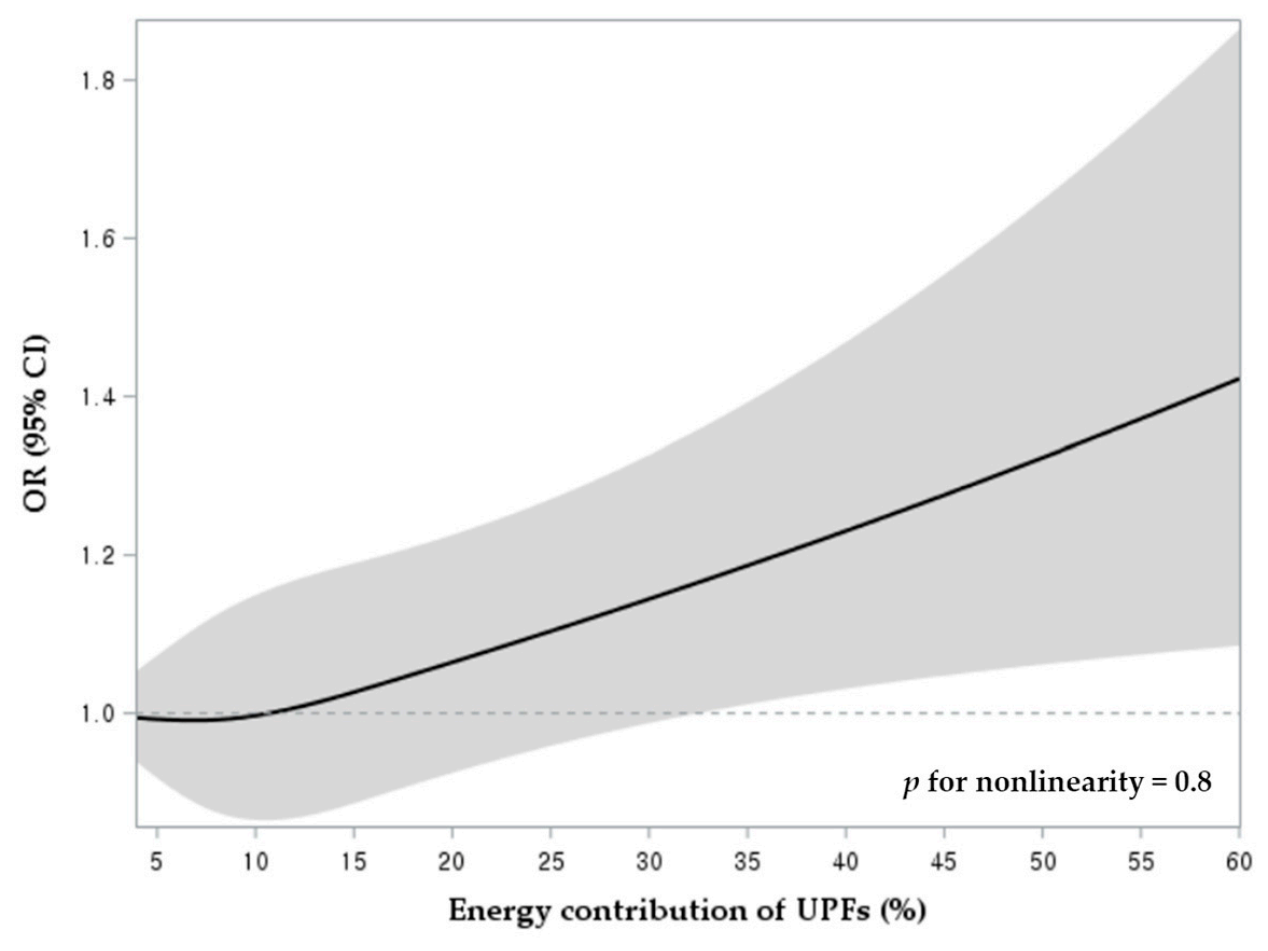
Restricted cubic spline analysis of the association between UPF energy contribution and frailty among Korean adults aged 65 years or older. The solid line represents adjusted odds ratios, and the shaded area represents 95% confidence intervals. The model was adjusted for age, sex, household income, educational level, survey year, alcohol consumption, smoking status, body mass index, and total energy intake. UPF, ultra-processed food; OR, odds ratio; CI, confidence interval.

**Table 1:** Demographic and lifestyle characteristics according to quartiles of UPF energy contribution among Korean adults aged 65 years or older, KNHANES 2014−2019, 2022 and 2024 (N = 9861).

Characteristics	UPF Energy Contribution				*p* Value
	Q1	Q2	Q3	Q4	
No. of participants	2465	2465	2466	2465	
UPF energy contribution (%)					
Mean ± SE	1.24 ± 0.02	5.47 ± 0.03	11.52 ± 0.06	29.26 ± 0.31	
Median (P25–P75)	1.08 (0.29–2.12)	5.38 (4.28–6.66)	11.20 (9.44–13.53)	25.39 (20.11–34.54)	
Range (min–max)	0.00–3.14	3.15–7.97	7.98–16.28	16.29–99.62	
Age (years)	72.59 ± 0.12	72.61 ± 0.12	72.44 ± 0.13	72.32 ± 0.12	0.227
Sex					<0.001
Men	1015 (42.98)	1129 (47.57)	1122 (48.58)	1170 (49.32)	
Women	1450 (57.02)	1336 (52.43)	1344 (51.42)	1295 (50.68)	
Household income					0.029
Low	1166 (44.73)	1101 (42.13)	1063 (40.74)	1042 (40.24)	
Middle	1068 (44.69)	1133 (47.79)	1128 (47.39)	1128 (46.84)	
High	221 (10.59)	226 (10.08)	270 (11.87)	288 (12.92)	
Educational level					<0.001
Elementary school or lower	1464 (56.64)	1399 (54.83)	1332 (50.54)	1131 (44.08)	
Middle school	378 (15.51)	406 (17.23)	394 (16.42)	414 (16.17)	
High school	423 (19.02)	408 (17.42)	449 (19.75)	572 (24.13)	
College or higher	185 (8.83)	243 (10.52)	284 (13.29)	342 (15.61)	
Alcohol consumption					<0.001
Non-drinkers	1278 (51.08)	1134 (44.54)	1199 (47.49)	1175 (46.20)	
Drinkers	1187 (48.92)	1329 (55.46)	1266 (52.51)	1290 (53.80)	
Smoking status					<0.001
Non-current smokers	2305 (93.51)	2241 (90.51)	2244 (90.73)	2167 (87.86)	
Current smokers	160 (6.50)	221 (9.49)	222 (9.27)	298 (12.14)	
Body mass index (kg/m^2^)	24.17 ± 0.08	23.99 ± 0.07	24.18 ± 0.07	24.11 ± 0.07	0.204
Total energy intake (kcal/day)	1613.50 ± 17.13	1710.17 ± 15.45	1691.32 ± 16.61	1688.84 ± 15.48	<0.001

KNHANES, Korea National Health and Nutrition Examination Survey; UPF, ultra-processed food; Q, quartile; SE, standard error; P25, 25th percentile; P75, 75th percentile. Unless otherwise indicated, values are weighted means ± standard errors for continuous variables and unweighted *n* (weighted %) for categorical variables. Medians and the 25th and 75th percentiles are survey-weighted. Ranges for UPF energy contribution represent the observed minimum and maximum values within each quartile. *p* values were obtained using design-adjusted chi-square tests for categorical variables and survey-weighted linear regression for continuous variables. Participant counts vary across characteristics because of missing data.

**Table 2:** Odds ratios (95% confidence intervals) for frailty and its components according to quartiles of UPF energy contribution among Korean adults aged 65 years or older, KNHANES 2014−2019, 2022 and 2024 (N = 9861).

Frailty Outcomes	UPF Energy Contribution				*p* for Trend
	Q1	Q2	Q3	Q4	
Frailty					
Cases, n (weighted %)	433 (15.73)	412 (15.81)	428 (16.12)	432 (17.10)	
Model 1	Ref	1.01 (0.84–1.21)	1.03 (0.87–1.21)	1.11 (0.93–1.32)	0.206
Model 2	Ref	1.03 (0.86–1.24)	1.09 (0.92–1.29)	1.20 (1.00–1.44)	0.040
Model 3	Ref	1.00 (0.82–1.21)	1.10 (0.92–1.31)	1.31 (1.08–1.59)	0.002
Unintentional weight loss					
Cases, n (weighted %)	273 (10.98)	284 (11.38)	276 (11.22)	280 (10.99)	
Model 1	Ref	1.04 (0.84–1.28)	1.02 (0.83–1.26)	1.00 (0.81–1.23)	0.880
Model 2	Ref	1.04 (0.84–1.28)	1.03 (0.84–1.28)	1.02 (0.82–1.25)	0.990
Model 3	Ref	1.07 (0.86–1.33)	1.11 (0.89–1.39)	1.06 (0.85–1.32)	0.691
Weakness					
Cases, n (weighted %)	894 (34.59)	863 (34.25)	825 (31.01)	839 (33.64)	
Model 1	Ref	0.99 (0.85–1.14)	0.85 (0.74–0.98)	0.96 (0.83–1.10)	0.521
Model 2	Ref	1.00 (0.86–1.16)	0.87 (0.76–1.01)	1.02 (0.88–1.19)	0.793
Model 3	Ref	0.95 (0.81–1.11)	0.86 (0.74–1.00)	1.10 (0.94–1.28)	0.117
Slowness					
Cases, n (weighted %)	905 (35.50)	866 (33.73)	910 (35.18)	851 (33.43)	
Model 1	Ref	0.93 (0.81–1.06)	0.99 (0.86–1.13)	0.91 (0.79–1.05)	0.314
Model 2	Ref	0.95 (0.82–1.09)	1.04 (0.90–1.20)	0.97 (0.84–1.13)	0.912
Model 3	Ref	0.97 (0.83–1.12)	1.08 (0.93–1.25)	1.03 (0.89–1.21)	0.495
Exhaustion					
Cases, n (weighted %)	415 (16.58)	389 (15.70)	391 (15.34)	380 (14.88)	
Model 1	Ref	0.94 (0.79–1.12)	0.91 (0.77–1.08)	0.88 (0.74–1.05)	0.178
Model 2	Ref	0.97 (0.81–1.16)	0.95 (0.80–1.12)	0.92 (0.77–1.09)	0.325
Model 3	Ref	0.96 (0.80–1.15)	0.96 (0.80–1.14)	0.97 (0.81–1.16)	0.824
Low physical activity					
Cases, n (weighted %)	845 (31.47)	882 (33.52)	947 (35.95)	888 (34.51)	
Model 1	Ref	1.10 (0.96–1.26)	1.22 (1.07–1.40)	1.15 (1.00–1.32)	0.090
Model 2	Ref	1.11 (0.97–1.27)	1.25 (1.09–1.44)	1.19 (1.03–1.36)	0.032
Model 3	Ref	1.12 (0.98–1.28)	1.32 (1.14–1.52)	1.31 (1.13–1.51)	0.001

The survey-weighted percentages of robust participants in Q1–Q4 were 29.80%, 30.06%, 29.77%, and 31.73%, respectively; the corresponding percentages of prefrail participants were 54.47%, 54.12%, 54.11%, and 51.17%. KNHANES, Korea National Health and Nutrition Examination Survey; UPF, ultra-processed food; Q, quartile. Case numbers are unweighted; percentages are survey-weighted. Model 1: Unadjusted. Model 2: Adjusted for age and sex. Model 3: Model 2 plus household income, educational level, survey year, alcohol consumption, smoking status, body mass index and total energy intake.

## Data Availability

The datasets supporting the conclusions of this article are available from the Korea Disease Control and Prevention Agency on reasonable request. These datasets are available with permission at the following URL: https://knhanes.kdca.go.kr/knhanes/rawDataDwnld/rawDataDwnld.do (accessed on 29 June 2026).

## Supplementary Materials

The following supporting information can be downloaded at https://www.mdpi.com/article/10.3390/nu18193135/s1. Supplementary Figure S1: Flowchart of participant selection for the primary frailty analysis; Supplementary Figure S2: Subgroup analyses of the association between UPF energy contribution and frailty among Korean older adults; Supplementary Table S1: Classification of ultra-processed foods and examples reported in previous studies using KNHANES data; Supplementary Table S2: Specific foods and beverages included in each ultra-processed food category; Supplementary Table S3: Annual weighted mean percentage of total energy intake from ultra-processed foods among Korean adults aged ≥65 years, KNHANES 2014–2024; Supplementary Table S4: Odds ratios (95% confidence intervals) for frailty comparing the highest versus lowest quartile of UPF energy contribution after separate adjustment for individual covariates; Supplementary Table S5: Odds ratios (95% confidence intervals) for frailty according to quartiles of UPF energy contribution using alternative definitions of low physical activity; Supplementary Table S6: Sensitivity analysis of frailty and its components after excluding industrially manufactured fermented sauces from the UPF classification; Supplementary Table S7: Number of consumers and energy intake from ultra-processed food categories among Korean older adults aged ≥65 years. References [37,47,51,52,53,54,55,56,57,58,59,60] are cited in the Supplementary Materials.

## Abbreviations

The following abbreviations are used in this manuscript:

UPFs	ultra-processed foods
KNHANES	Korea National Health and Nutrition Examination Survey
EQ-5D	EuroQol-5 Dimensions
IRB	Institutional Review Board
BMI	body mass index
ORs	odds ratios
CIs	confidence intervals
