# Supplementary material for: Association Between Ultra-Processed Food Consumption and Frailty in Korean Older Adults
Source: Nutrients. 2026 Sep 23;18(19):3135. doi: 10.3390/nu18193135 (PMC13667227; doi:10.3390/nu18193135)
Supplement: Supplementary file 1 [file nutrients-18-03135-s001.zip › nutrients-4557992-supplementary.pdf]

Supplementary materials

Association between ultra-processed food consumption and frailty among Korean older adults

Hyewon Lee & Kyong Park

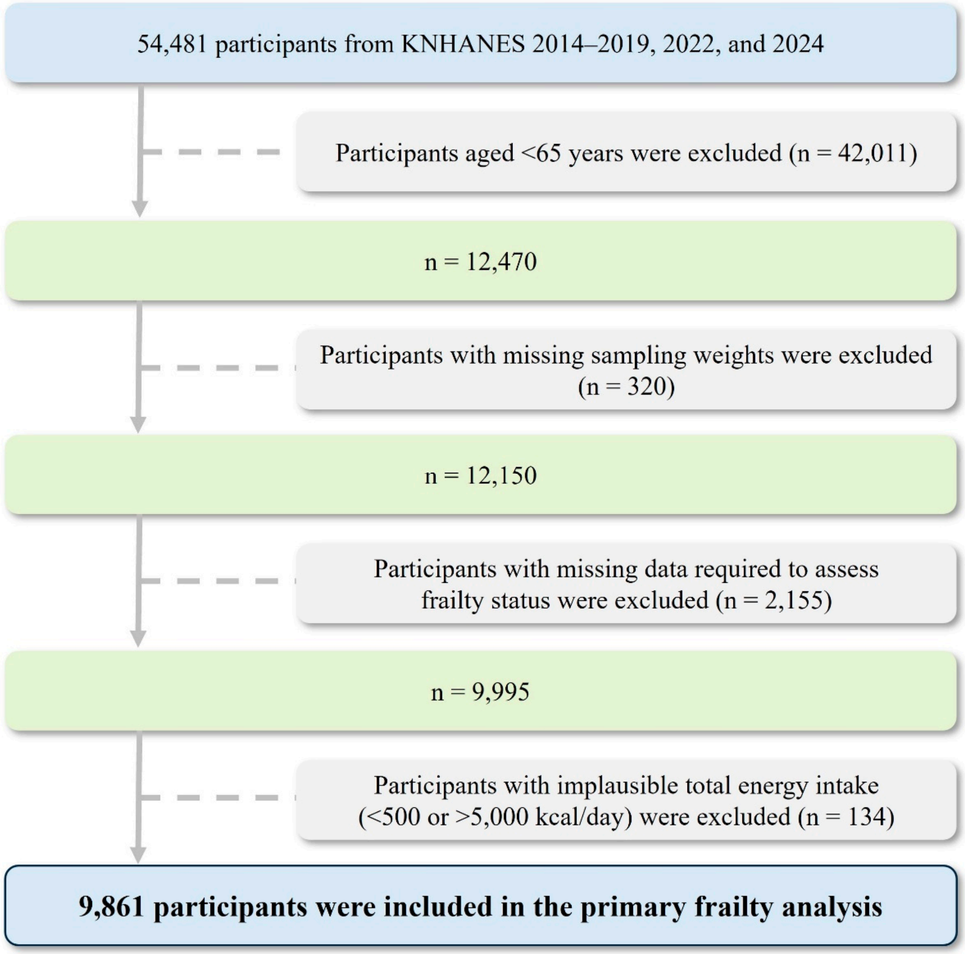

Supplementary Figure S1. Flowchart of participant selection for the primary frailty analysis. KNHANES, Korea National Health and Nutrition Examination Survey. Survey years were restricted to those in which all variables required to assess frailty were available.

Supplementary Table S1. Classification of ultra-processed foods and examples reported in previous studies using KNHANES data

Author (year)	Age (years)	Mean UPF intake (%kcal)	Examples of foods classified as UPFs
1. Jung *et al.* (2024) [37]	≥40	26.8	• Bread<br>• Chocolate milk<br>• Sausage
2. Jung *et al.* (2024) [51]	20–69	24.2	• Soy sauce (manufactured), soybean paste, red pepper paste (manufactured), ssamjang<br>• MSG, anchovy broth<br>• Plum syrup<br>• Coffee mix, coffee creamer<br>• Ketchup, mayonnaise<br>• Bread crumbs, bread, snacks<br>• Coke, yogurt drink, soju, distilled alcoholic beverages, soft drinks<br>• Noodles, glass noodle, fried fishcake
3. Park *et al.* (2024) [52]	≥19	22.0	• Beverages (soda, fruit, and vegetable drinks)<br>• Bread, cakes, and bakery products<br>• Sauce, dressing, and condiments<br>• Instant noodles<br>• Processed fish and meat products<br>• Distilled alcoholic drinks<br>• Cookies, chips, and snacks<br>• Ready-to-eat products<br>• Frozen foods (pizza, hamburger, hotdog)<br>• Confectionery
4. Kim *et al.* (2024) [53]	≥19	20.3	• Fatty, sweet, savory, or salty packaged snacks, biscuits (cookies)<br>• Ice creams and frozen desserts<br>• Chocolates, candies, and confectionery in general<br>• Cola, soda, and other carbonated soft drinks<br>• ‘Energy’ and sports drinks<br>• Canned, packaged, dehydrated (powdered), and other ‘instant’ soups, noodles, sauces, desserts, drink mixes, and seasonings<br>• Sweetened and flavored yogurts, including fruit yogurts<br>• Dairy drinks, including chocolate milk<br>• Sweetened juices<br>• Margarine and spreads<br>• Pre-prepared (packaged) meat, fish, poultry, vegetables, pizza, pasta dishes, burgers, hot dogs, sausages, nuggets<br>• Other animal products made from remnants<br>• Packaged bread<br>• Baked products made with ingredients such as hydrogenated vegetable fat, sugar, yeast, whey, emulsifiers, and other additives<br>• Breakfast cereals and bars<br>• Baby formulas, drinks, and meal replacement shakes (e.g., ‘slim fast’)<br>• Pastries, cakes, and cake mixes<br>• Distilled alcoholic beverages such as whisky, gin, rum, vodka.<br>• Margarines, peanut butter, all syrups (excluding processed culinary ingredients), artificial sweeteners, and Korean fermented condiments’ added food additives
5. Jung et *al.* (2023) [54]	≥40	24.2	• Mass-produced bread, snacks, soft drinks, and noodles
6. Jung *et al.* (2022) [55]	≥19	32.8	• Bread (mass-produced packaged); biscuits; cakes; cookies; chocolate; candies; ice cream<br>• Carbonated or fruit drinks<br>• Pre-prepared dishes, sausages; hot dogs, burgers, nuggets, and instant soups, noodles or dumplings

7. Shim *et al.* (2022) [56]	≥19	26.2	• Sugar-sweetened beverages<br>• Soft drinks, fruit and vegetable drinks<br>• Sweetened milk and its products<br>• Cereals, breads, cakes, sandwiches, etc.<br>• Traditional sauce<br>• Confectionary<br>• Others (instant sauce, condiments, etc.)<br>• Cookies, chips, and snacks<br>• Fish and meat processed foods<br>• Instant noodles and dumplings<br>• Instant cooked rice, soup, and other dishes<br>• Distilled alcoholic beverages
8. Shim *et al.* (2022) [57]	30–79	23.6	• Breakfast cereals, breads and cakes, cookies, sweet or salty snacks, candies, chocolates, desserts, ice cream<br>• Sugary milk and fruit and vegetable drinks, soft drinks, sugared teas and coffee, distilled alcoholic beverages<br>• Meat and fish products (e.g. chicken nuggets)<br>• Instant sauces and spreads, sweeteners<br>• Instant formulas and baby products<br>• Diverse sorts of ready-to-eat or ready-to-heat foods (e.g., instant rice, noodles, soups, dumplings, or pizza)
9. Shim *et al.* (2022) [47]	≥19	25.1	• Cereals, breads, cakes, sandwiches, etc.<br>• Distilled alcoholic beverages<br>• Sugar-sweetened beverages<br>• Fish and meat processed foods<br>• Instant noodles and dumplings<br>• Traditional sauce<br>• Sweetened milk and its products<br>• Others (instant sauce, condiments, etc.)<br>• Cookies, chips, and snacks<br>• Soft drinks, fruit and vegetable drinks<br>• Instant cooked rice, soup, and other dishes<br>• Confectionary
10. Park *et al.* (2022) [58]	≥19	29.3	• Candy, chewing gum<br>• Mix & beverage<br>• Peanut butter<br>• Ground processed meat, processed ham<br>• Processed cheese
11. Shim *et al.* (2021) [59]	≥1	24.9	• Breakfast cereals<br>• Breads and cakes<br>• Cookies<br>• Sweet or salty snacks<br>• Candies and chocolates<br>• Desserts and ice cream<br>• Sugary milk drinks<br>• Sugary fruit and vegetable drinks<br>• Soft drinks<br>• Sugared teas and coffee<br>• Distilled alcoholic beverages<br>• Meat and fish products (e.g., chicken nuggets)<br>• Instant sauces and spreads<br>• Sweeteners<br>• Instant formulas and baby products<br>• Ready-to-eat or ready-to-heat foods (e.g., instant rice, noodles, soups, dumplings, or pizza)

12. Sung *et al.* (2021) [60]	19–64	26.8	• Beverages (instant coffee and coffee drinks, carbonated drinks, fruit drinks, and other sugar added drinks)<br>• Bread, cakes, and bakery products<br>• Sauce, dressing, and condiments (includes margarines, peanut butter, all syrups (excluding processed culinary ingredients), artificial sweeteners, and Korean fermented condiments added food additives)<br>• Instant noodles<br>• Distilled alcoholic drinks<br>• Meat and seafood products (includes ham, sausages, chicken nuggets, fried chicken, and Japanese fish cakes)<br>• Sweet/salty snacks<br>• Milk-based drinks, processed cheese, and ice cream<br>• Ready to eat/heat products and other home meal replacements<br>• Fast foods (hamburger, pizza, and hotdog)<br>• Confectionary, jam, and ice pops<br>• Breakfast cereals<br>• Others (includes health and weight control food, and baby formula)

KNHANES, Korea National Health and Nutrition Examination Survey; UPF, ultra-processed food.
Mean UPF intake values were those reported in the original studies and may not be directly comparable because of differences in study populations, survey years, and UPF classification methods.

Supplementary Table S2. Specific foods and beverages included in each ultra-processed food category

**Ultra-processed food category**	**Specific foods and beverages**
1. Packaged breads, breakfast cereals, sweet or savory snacks, and desserts	Packaged breads and buns; breakfast cereals; confectionery products (e.g., chocolates, candies/ caramels, bars, jellies); packaged sweet and savory snacks and desserts; dairy-based processed products (e.g., ice cream, ice pops, sherbet/sorbet, flavored yogurts, and processed cheese)
2. Convenience foods and animal-based processed foods	Pizza; instant soups (powdered or packaged); packaged instant noodles (e.g., *ramyeon, udon, soba*); packaged sandwiches; French fries and hash browns; dumplings; grain-based and Asian-style mixed dishes (e.g., frozen, microwavable, prepared, canned, or boxed instant cooked rice dishes; dishes containing non-culinary ingredients); hamburgers; hot dogs; croquettes; retort-packaged jjajang sauce/powder and curry sauce/roux/powder; sausages; ham; bacon; fish cakes; crab sticks; meatballs; poultry and fish nuggets
3. Artificially sweetened and sugar-sweetened beverages	Carbonated soft drinks (e.g., cola, cider, flavored or sweetened sparkling water, carbonated fruit-flavored soft drinks); industrially formulated fruit, vegetable, and grain drinks (e.g., apple, orange, grape, plum, pear, mandarin, peach, grapefruit, lime, tomato, and carrot juices; rice punch); chocolate/cocoa drinks; coffee products containing added sugar, creamer, flavorings, or other additives; sweetened or flavored milk drinks (e.g., strawberry-, banana-, chocolate-, and coffee-flavored milk; milkshakes); soy milk drinks; health and slimming products (e.g., meal replacement shakes, nutrition supplement powders and drinks); sports drinks; energy drinks; infant formula and follow-on formula
4. Sauces, condiments, and spreads	Salad dressings; industrially manufactured fermented sauces containing non-culinary ingredients or food additives (e.g., *doenjang*, *soy sauce*, *gochujang*, and *ssamjang*); powdered seasonings (e.g., *Dashida* (Korean beef stock powder), anchovy extract, beef extract); other seasoning sauces (e.g., ketchup, mayonnaise, mustard, ramyeon soup, meat dipping sauce, hot sauce, red chili sauce); margarine; peanut butter

Supplementary Table S3. Annual weighted mean percentage of total energy intake from ultra-processed foods among Korean adults aged ≥65 years, KNHANES 2014–2024

Survey year	Primary frailty analyses, n	UPF temporal-trend analysis, n	UPF energy contribution (% of total energy)	
			Weighted Mean	95% CI
2014	1036	1437	11.10	10.39-11.81
2015	1037	1356	11.10	10.43-11.77
2016	1099	1356	12.47	11.72-13.22
2017	1316	1464	12.07	11.34-12.81
2018	1294	1428	11.82	11.14-12.50
2019	1328	1512	12.49	11.83-13.14
2020	-	1339	12.90	12.13-13.68
2021	-	1575	13.77	12.96-14.59
2022	1234	1537	12.36	11.62-13.11
2023	-	1770	13.10	12.42-13.78
2024	1517	1871	12.52	11.97-13.06
Total	9861	16,645	12.42	12.21-12.63

Participant counts (n) are unweighted.
Weighted means and 95% CIs were estimated using survey weights for the UPF temporal-trend sample.
The primary frailty analysis additionally required complete frailty data; the 2020–2021 survey years were excluded because handgrip strength data were unavailable, and 2023 was excluded because EQ-5D data were unavailable.
KNHANES, Korea National Health and Nutrition Examination Survey; UPF, ultra-processed food; CI, confidence interval; EQ-5D, EuroQol 5-Dimension.

Supplementary Table S4. Odds ratios (95% confidence intervals) for frailty comparing the highest versus lowest quartile of UPF energy contribution after separate adjustment for individual covariates

Model/adjustmnet	Covariates included	OR (95% CI)
Model 2	Age, sex	1.20 (1.00-1.44)
Model 2 + household income	Age, sex, household income	1.21 (1.01-1.45)
Model 2 + educational level	Age, sex, educational level	1.36 (1.13-1.63)
Model 2 + survey year	Age, sex, survey year	1.20 (1.00-1.44)
Model 2 + alcohol consumption	Age, sex, alcohol consumption	1.21 (1.01-1.46)
Model 2 + smoking status	Age, sex, smoking status	1.17 (0.98-1.41)
Model 2 + BMI	Age, sex, BMI	1.18 (0.98-1.43)
Model 2 + total energy intake	Age, sex, total energy intake	1.21 (1.01-1.46)
Model 3	Age, sex, household income, educational level, survey year, alcohol consumption, smoking status, BMI, and total energy intake	1.31 (1.08-1.59)

ORs and 95% CIs were estimated using survey-weighted logistic regression. Each intermediate model included age and sex plus one additional covariate, as indicated, to examine changes in the Q4 versus Q1 estimate after adjustment for each individual covariate.

UPF, ultra-processed food; Q, quartile; OR, odds ratio; CI, confidence interval; BMI, body mass index.

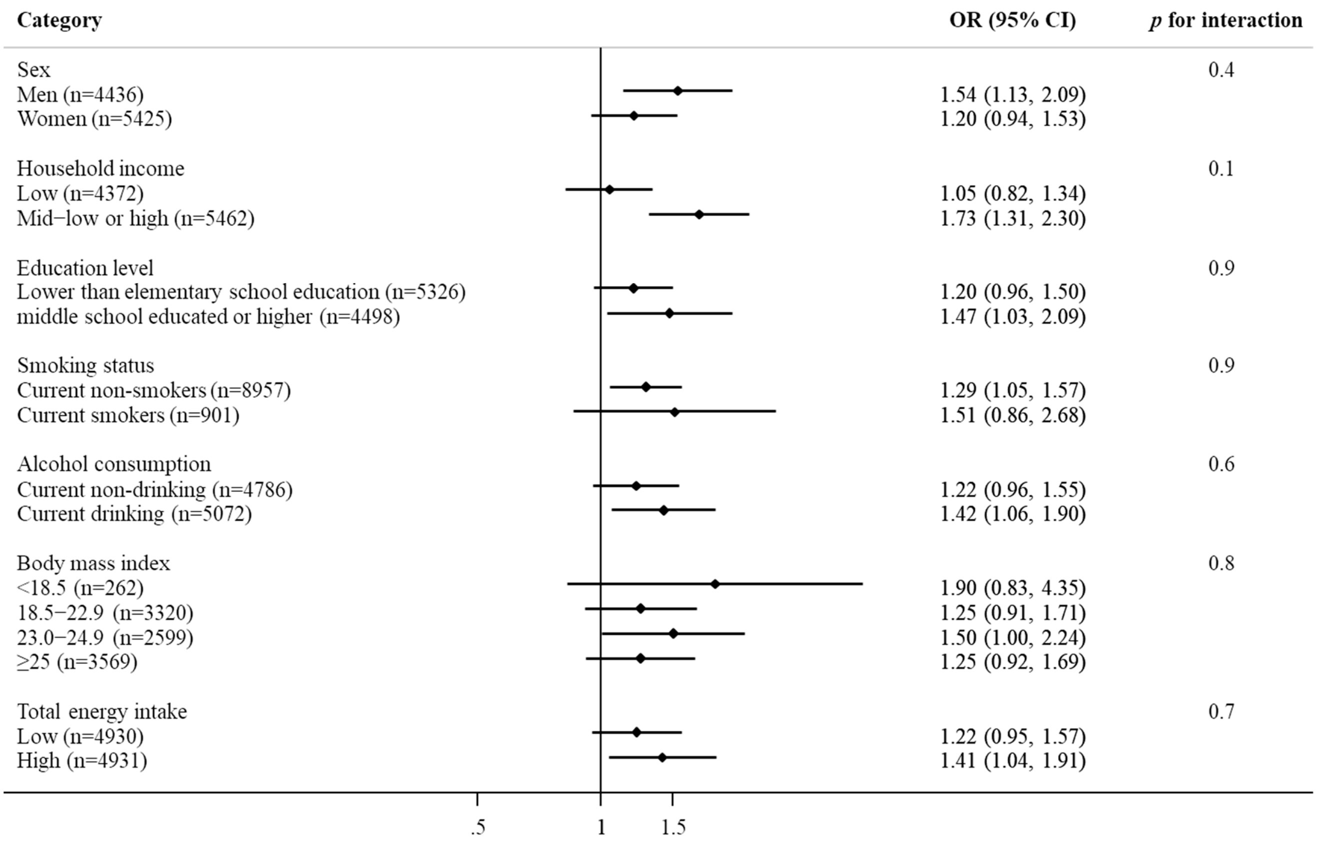

Supplementary Figure S2. Subgroup analyses of the association between UPF energy contribution and frailty among Korean older adults. ORs and 95% CIs compare Q4 with Q1 and were adjusted for Model 3 covariates, except the stratification variable. UPF, ultra-processed food; OR, odds ratio; CI, confidence interval. Subgroup sample sizes may not sum to the total sample size because of missing data. UPF, ultra-processed food; OR, odds ratio; CI, confidence interval.

Supplementary Table S5. Odds ratios (95% confidence intervals) for frailty according to quartiles of UPF energy contribution using alternative definitions of low physical activity

Low physical activity definition	UPF energy contribution				*p* for Trend
	Q1	Q2	Q3	Q4	
Moderate/vigorous activity-based definition[1] (n = 9867)					
No. of participants	2466	2467	2467	2467	
Frailty cases, n (weighted %)	698 (27.06)	679 (26.50)	672 (25.43)	656 (25.94)	
Model 1	Ref	0.97 (0.84-1.13)	0.92 (0.80-1.06)	0.94 (0.81-1.10)	0.466
Model 2	Ref	1.00 (0.86-1.16)	0.96 (0.83-1.12)	1.01 (0.87-1.18)	0.880
Model 3	Ref	0.98 (0.83-1.14)	0.98 (0.84-1.14)	1.08 (0.92-1.27)	0.225
Walking-based definition[2] (n = 9802)					
No. of participants	2450	2451	2451	2450	
Frailty cases, n (weighted %)	458 (16.63)	436 (16.90)	453 (17.09)	462 (18.22)	
Model 1	Ref	1.02 (0.86-1.22)	1.03 (0.88-1.22)	1.12 (0.94-1.33)	0.175
Model 2	Ref	1.05 (0.88-1.26)	1.10 (0.93-1.31)	1.22 (1.02-1.46)	0.024
Model 3	Ref	1.01 (0.84-1.22)	1.12 (0.94-1.33)	1.33 (1.11-1.61)	0.001

Case numbers are unweighted; percentages are survey-weighted. ORs and 95% CIs were estimated using survey-weighted logistic regression.
Model 1: Unadjusted.
Model 2: Adjusted for age and sex.
Model 3: Model 2 plus household income, educational level, survey year, alcohol consumption, smoking status, body mass index, and total energy intake.
[1] Low physical activity was defined as <120 min/week of moderate-intensity activity or <60 min/week of vigorous-intensity activity.
[2] Low physical activity was defined as <120 min/week of walking.
UPF, ultra-processed food; Q, quartile.

Supplementary Table S6. Sensitivity analysis of frailty and its components after excluding industrially manufactured fermented sauces from the UPF classification

Frailty outcomes	UPF energy contribution				*p* for Trend
	Q1	Q2	Q3	Q4	
Frailty					
Cases, n (weighted %)	461 (17.39)	381 (14.25)	435 (16.20)	428 (16.98)	
Model 1	Ref	0.79 (0.66-0.95)	0.92 (0.77-1.09)	0.97 (0.82-1.15)	0.440
Model 2	Ref	0.84 (0.70-1.01)	1.00 (0.83-1.19)	1.06 (0.89-1.27)	0.120
Model 3	Ref	0.89 (0.73-1.07)	1.07 (0.89-1.28)	1.25 (1.03-1.51)	0.002
Unintentional weight loss					
Cases, n (weighted %)	291 (11.80)	259 (10.34)	286 (11.86)	277 (10.61)	
Model 1	Ref	0.86 (0.69-1.08)	1.01 (0.82-1.24)	0.89 (0.72-1.09)	0.488
Model 2	Ref	0.88 (0.70-1.09)	1.03 (0.83-1.26)	0.91 (0.74-1.12)	0.607
Model 3	Ref	0.94 (0.75-1.18)	1.11 (0.89-1.38)	0.98 (0.79-1.22)	0.999
Weakness					
Cases, n (weighted %)	912 (35.30)	850 (33.45)	827 (31.35)	832 (33.41)	
Model 1	Ref	0.92 (0.80-1.07)	0.84 (0.73-0.97)	0.92 (0.80-1.06)	0.392
Model 2	Ref	0.98 (0.85-1.14)	0.89 (0.77-1.03)	1.00 (0.86-1.16)	0.963
Model 3	Ref	1.03 (0.88-1.20)	0.93 (0.80-1.09)	1.14 (0.98-1.34)	0.073
Slowness					
Cases, n (weighted %)	931 (37.14)	844 (32.62)	902 (34.50)	855 (33.62)	
Model 1	Ref	0.82 (0.71-0.94)	0.89 (0.78-1.02)	0.86 (0.75-0.99)	0.196
Model 2	Ref	0.87 (0.75-1.01)	0.95 (0.83-1.10)	0.92 (0.79-1.07)	0.636
Model 3	Ref	0.94 (0.81-1.10)	1.01 (0.87-1.17)	1.02 (0.87-1.19)	0.507
Exhaustion					
Cases, n (weighted %)	435 (17.36)	370 (14.97)	390 (15.18)	380 (15.01)	
Model 1	Ref	0.84 (0.70-1.00)	0.85 (0.72-1.01)	0.84 (0.71-1.00)	0.171
Model 2	Ref	0.88 (0.73-1.05)	0.88 (0.74-1.05)	0.87 (0.73-1.03)	0.256
Model 3	Ref	0.88 (0.73-1.06)	0.91 (0.76-1.08)	0.94 (0.79-1.12)	0.875
Low physical activity					
Cases, n (weighted %)	871 (32.57)	839 (32.21)	962 (36.16)	890 (34.56)	
Model 1	Ref	0.98 (0.86-1.13)	1.17 (1.03-1.34)	1.09 (0.96-1.25)	0.123
Model 2	Ref	1.01 (0.89-1.16)	1.22 (1.07-1.40)	1.14 (0.99-1.31)	0.046
Model 3	Ref	1.07 (0.93-1.23)	1.31 (1.15-1.51)	1.30 (1.13-1.50)	<0.001

Case numbers are unweighted; percentages are survey-weighted. ORs and 95% CIs were estimated using survey-weighted logistic regression. UPF energy contribution and quartiles were recalculated after excluding industrially manufactured fermented sauces.
Model 1: Unadjusted.
Model 1: Unadjusted.
Model 2: Adjusted for age and sex.
Model 3: Model 2 plus household income, educational level, survey year, alcohol consumption, smoking status, body mass index, and total energy intake.
UPF, ultra-processed food; Q, quartile.

Supplementary Table S7. Number of consumers and energy intake from ultra-processed food categories among Korean adults aged ≥65 years

UPF category	Consumers n (weighted %)[1]	Energy intake (kcal/day)	
		Mean	Range (min−max)
Total ultra-processed foods	9650 (98.02)	204.28	0.00-3618.11
Packaged breads, breakfast cereals, sweet or savory snacks, and desserts	3313 (34.71)	65.76	0-2306.72
Packaged breads and buns	1092 (11.72)	25.09	0.00-1747.04
Packaged sweet and savory snacks and desserts	1363 (14.31)	23.59	0.00-1503.09
Dairy-based processed products [2]	900 (9.66)	10.90	0.00-639.00
Confectionery products [3]	695 (6.73)	4.74	0.00-935.79
Breakfast cereals	78 (0.83)	1.43	0.00-582.81
Convenience foods and animal-based processed foods	2373 (24.67)	45.87	0.00-2395.36
Instant noodles	700 (7.31)	26.97	0.00-1567.10
Ready-to-eat or ready-to-heat dishes [4]	386 (4.18)	10.15	0.00-2395.36
Processed meat, seafood, and poultry products [5]	1306 (13.53)	6.56	0.00-686.37
Instant soups, curries, and jjajang products	320 (3.33)	2.19	0.00-972.80
Artificially sweetened and sugar-sweetened beverages	6022 (61.29)	57.63	0.00-2556.00
Instant cocoa and coffee drinks	4664 (47.05)	34.66	0.00-2556.00
Milk- and soy milk-based drinks	855 (8.68)	13.64	0.00-863.28
Fruit, vegetable, and grain drinks	1112 (11.46)	5.21	0.00-399.36
Carbonated soft drinks	568 (5.86)	2.44	0.00-272.27
Other drinks [6]	230 (2.37)	1.68	0.00-977.14
Sauces, condiments, and spreads	9096 (92.73)	35.02	0.00-1313.69
Industrially manufactured fermented sauces [7]	8582 (87.45)	26.13	0.00-627.99
Other seasoning sauces [8]	1268 (13.42)	7.50	0.00-1278.59
Powdered seasonings [9]	5936 (61.32)	0.62	0.00-46.48
Margarine and peanut butter	123 (1.24)	0.37	0.00-611.56
Salad dressings	61 (0.78)	0.40	0.00-312.99

UPF, ultra-processed food.
Consumer counts (n) are unweighted; percentages and mean energy intakes are survey-weighted.
Range indicates minimum–maximum values and is not weighted.
Mean energy intake was calculated among all participants, including non-consumers.
[1] Consumers were defined as participants with intake > 0 kcal.
[2] Includes ice creams, ice pops, sherbet/sorbet, flavored yogurts and processed cheese.
[3] Includes chocolates, candies, caramels, bars and jellies.
[4] Includes pizza, packaged sandwiches, French fries and hash browns, dumplings, hamburgers, hot dogs, croquettes and grain-based and Asian-style mixed dishes (e.g., frozen, microwavable, prepared, canned, or boxed instant cooked rice dishes; dishes containing non-culinary ingredients).
[5] Includes ham, bacon, fish cakes, crab sticks, meatballs and poultry/fish nuggets.
[6] Includes ‘health’ and ‘slimming’ products (e.g., meal replacement shakes, nutrition supplement powders and drinks), sports drinks, energy drinks, infant formula and follow-on formula.
[7] Includes industrially manufactured *doenjang, gochujang, ssamjang* and soy sauce.
[8] Includes ketchup, mayonnaise, mustard, *ramyeon* soup, meat dipping sauce, hot sauce and red chili sauce.
[9] Includes *Dashida*, anchovy extract and beef extract.
